# The Use of Lausanne Trilogue Play in Three Cases of Gastroschisis Diagnosed during Pregnancy

**DOI:** 10.3389/fpsyg.2017.00509

**Published:** 2017-04-03

**Authors:** Sandra Pellizzoni, Antonella Tripani, Marina Miscioscia, Rosella Giuliani, Andrea Clarici

**Affiliations:** ^1^Department of Life Science, University of TriesteTrieste, Italy; ^2^Scricciolo – Associazione Genitori di Bambini Nati Prematuri o a Rischio c/o Institute for Maternal and Child Health – IRCCS “Burlo Garofolo,"Trieste, Italy; ^3^Department of Developmental and Social Psychology, University of PaduaPadua, Italy; ^4^ABC Associazione per i Bambini Chirurgici del Burlo Onlus c/o Institute for Maternal and Child Health – IRCCS “Burlo Garofolo,"Trieste, Italy; ^5^Department of Medical, Surgical and Health Science, University of TriesteTrieste, Italy

**Keywords:** prenatal diagnosis, Lausanne trilogue play, parents, gastroschisis, fetal malformations

## Abstract

From pregnancy to the 1st years of a child’s life, families develop and increase representations and interactive competences toward the child. Prenatal diagnosis of a severe fetus’ defect could profoundly alter the parental perception and development of these representations. The aim of the study was to evaluate triadic interactions in families, whose baby was prenatally diagnosed with severe gastroschisis. Three families took part in the preliminary case study, which was carried out when the babies were 6 months old. The Lausanne Trilogue Play shows that prenatal diagnosis of fetal malformation may affect family triadic interactions as follows: (a) parents, especially mothers, tend to be intrusive during the play; (b) parents presents maladjustments in the child stimulations, especially during the third part, when both parents have to simultaneously interact with the baby; (c) parents experience difficulties in creating a space that allows them to communicate directly with each other, leaving the child in a peripheral position. Observational data and clinical implications are discussed.

## Introduction

Gastroschisis is a birth defect affecting the intestinal wall, which results in the intra-amniotic extrusion of parts of the fetus’ intestine. Gastroschisis is marked by an elevated degree of severity, increased incidence registered in recent years (Feldkamp et al., 2007), the early stage at which it is detected, the high level of medicalisation during pregnancy and postpartum period, the fact that younger mothers are at greater risk (Williams et al., 2005), and the long-term consequences on the patient’s conditions caused by the defect. The simultaneous presence of several risk factors led us to organize a project linking experimental observation and support for parents, in order to learn more about the possible effects of the illness on early interactions in families having to deal with this condition.

Prenatal diagnosis of any fetus defect profoundly alters the perception of pregnancy and its representations (Tripani et al., 2015). Empirical research has shown that parents’ intra-psychic dynamics during the perinatal period have an impact on the development of representations and interactive capabilities in the months following birth (Siddiqui and Hägglöf, 2000). Numerous studies in the field highlighted the importance of triadic family interaction as one of the most relevant factors in structuring the baby’s psychosocial development (Parke and Buriel, 1998) in various salient aspects, such as attachment (Erdman and Caffery, 2003), school adjustment (McHale and Rasmussen, 1998), psychopathological development (Jacobvitz et al., 2004; Clarici et al., 2015; Gatta et al., 2017), and theory of mind (Favez et al., 2012). However, studies on the influence of defect diagnosis on parental capacity in the time span between pregnancy and postpartum period are still very scarce (Skari et al., 2006; Giuliani et al., 2014).

LTP is an instrument allowing for the observation of triadic interaction dynamics within a family through the analysis of relational capabilities and resources, as well as limits of interactive abilities that parents and children display when engaging in a semi-structured play activity. This play activity distinguishes between four relational configurations: (I) one parent interacts with the baby, while the other is present, but passive; (II) same as (I), but with roles reversed; (III) parents and baby play together; (IV) parents interact with each other in the presence of the baby. Families’ interactions were filmed using cameras oriented toward the family; recordings were subsequently analyzed, using the FAAS coding system (Family Alliance Assessment Scale 6.3; Lavanchy-Scaiola et al., unpublished). The FAAS coding system is based on 15 variables that evaluate the construct of Family Alliance. Each LTP variable coded with a three-point Likert scale: 0 (inappropriate), 1 (moderate), 2 (appropriate). The total score can range from 0 to 30. Analysis of the clinical cases reported below was carried out using all 15 LTP variables (e.g., the “Inclusion” variable measures whether and to what extent the parties engage in the interaction, and whether they take each other into account; the “Parental scaffolding” variable measures parental stimulation with respect to the child’s age and state) (Favez et al., 2011).

## Background

Three couples, three fathers (F1, F2, and F3) and three mothers (M1, M2, and M3) with mean age 30 and 22 years, respectively, took part in the research. Participants had a high-school diploma and were regularly employed. The families were informed about the malformation between the 14th and the 23rd week of pregnancy. This small sample was part of a more articulated research, which has recently been published (Giuliani et al., 2014; Tripani et al., 2015). Different types of evaluations were carried out before and after the babies’ birth; owing to that and the use of the camera during LTP observations only three consented take part in the research project. Furthermore, parents with previous diagnosis of mental illness were also excluded from the sample.

A psychotherapeutic psychologist assisted the families during the pregnancy and met the parents once a week during their stay at the pediatric hospital. The study was approved by the Ethics Committee of the Institute for Maternal and Child Health IRCCS “Burlo Garofolo” (Trieste, Italy) and every participant signed an informed consent before taking part in the research.

## Family 1: Medical History After B1’s Birth

M1 had a Cesarean section in the 34th week of pregnancy and B1 had to stay in neonatal intensive care for 89 days. At birth B1 weighed 1,830 g. After 2 h from birth, B1 underwent surgery to remove part of the intestines from its abdominal cavity, and it had a silo positioned for subsequent gradual reduction of the remaining intestines. After the operation B1 was put on mechanical ventilation for 6 days, after which he started breathing normally. B1’s intestine was completely reintegrated into the abdominal cavity on the child’s 10th day of life. A nasogastric tube ensured nutrition until the child was 1 month old. As intestinal function progressively improved, the evacuative enemas were gradually stopped, though there was still sporadic vomiting. B1 was eventually discharged in good conditions, weighing 4,230 g. When discharged, the child was being fed three meals of his mother’s milk from a feeding bottle, and five through the nasogastric tube, in continuous feeding lasting 3 h. Neither the child nor the parents, especially the mother, were ever able to adapt to the presence of the nasogastric tube.

## Family 1: LTP Procedure

M1 and F1 participate actively in the interaction, which is largely focused on B1. B1’s availability, however, is intermittent, as indicated by typical signals of deflection in facial expressions, and body and gaze slight rotations, with significant repercussions on the quality of the family’s relationship. When interacting with his mother, for instance, B1’s gaze wanders off, often away from the area of interaction, and he does not seem to be able to get involved, isolating himself for half of the observation time, despite M1 efforts to get his attention.

B1 is clearly interested in his dummy. He looks at it and tries to grasp it, without ever meeting his mother’s gaze. M1 moves B1 on his child seat, but he is uncomfortable and throws himself backward. The chosen stimuli are frequently unsuitable for the baby. When it is F1’s turn to interact with the baby, he is given the dummy and tries to catch B1’s attention with it, as M1 did. F1 does not speak as much as M1 did, he moves the dummy too close to the baby’s face, almost intrusively. B1 reacts by twisting his body away from the interaction space, while pulling the dummy toward himself. F1 lets go of his hold. Both parents appear to have some trouble touching the baby, and no game they play with him is structured. The dummy is essential, as it is the medium, through which their touch is mediated. Only when holding the dummy, the parents are able to touch B1.

As regards shared, co-constructed activities, partners participate in collective games, talking in turns, despite their inability to upgrade tasks and increment complexity. During the three-party interaction, very diverse activities are performed, none of which, however, is negotiated.

When parents are asked to interact with each other exclusively, they keep turning to B1, watching him and talking to him. Hardly any conversation topic does not involve their child and they are visibly uncomfortable.

M1 acknowledges B1’s experience, taking into account B1’s emotional displays; however, her reactions are not always appropriate. F1, on the other hand, only partially acknowledges B1’s experience, and, despite his noticing B1’s emotional displays during three-party interactions, he does not validate them. Parents’ emotional displays are almost forced, their smiles are embarrassed, and they are unable to interact naturally with B1, who tends to self-exclusion.

Despite their frequent silences, parents appear to support each other in facing difficulties. They tend, however, to focus on B1, keeping their gaze fixed on him. The Global Family Alliance level was assigned the medium-high score of 16.

## Family 2: Medical History After B2’s Birth

Immediately after the planned Cesarean section, B2 underwent surgery, during which its intestine was entirely inserted into the abdomen. B2 was born at 35 weeks, weighing 1,840 g. B2 was put on a tube 5 min after birth; its abdominal rupture was then manually reduced, and sterile dressing and tight bandages were applied. A nasogastric tube was then applied. On the 2nd day after birth, mechanical ventilation was suspended and B2 started breathing autonomously. On the 7th day after birth, feeding was resumed, and it kept improving gradually, until the child became completely autonomous at 2 weeks of age. B2 was kept in neonatal intensive care for 22 days, and was discharged with a weight of 2,250 g, feeding independently from the bottle or breast, on demand, approximately 70–80 ml every 3 h. The family was not required to follow any intrusive medical procedure.

## Family 2: LTP Procedure

As far as participation patterns are concerned, B2’s availability appears intermittent, as he turns his gaze repeatedly and at length away from the interaction space when playing with F2, and, slightly less frequently and more briefly, when playing with M2. During the three-party play session, B2 tends to self-exclude, probably due to a greater difficulty in self-regulating.

Disengagement from the interaction and/or expression of uneasiness break the game and the family atmosphere is mostly neutral. Both mother-child and father-child interactions, either during two-party and/or three-party activities, require numerous engagement efforts on the part of the baby. The child’s signals seem to be recognized and correctly interpreted by the parents, but their expectations appear to be too high with respect to the baby’s age.

Parental stimulation is frequently offset, as B2 is over stimulated with activities that require abilities beyond his reach, especially during three-party interaction, when both parents are clearly incapable of adapting to B2’s status. They verbalize their awareness of B2’s unwillingness to play, they say they can see that he is tired, but they still want him to do some “exercise.” During the first and second interaction phases (i.e., B2–F2 and B2–M2, respectively), B2’s parents succeed in playing games and complete sequences of speaking turns, despite their evident difficulty in bringing each activity forward; during three-party interactions, on the other hand, activities are carried out mostly on a one-to-one basis, rather than involving all three interactants at once. Co-construction is flawed: each couple plays intermittently, and only timidly extends the interaction to include the third party; gazes never embrace the entire activity circle; verbal elements are shared by parents exclusively; non-verbal elements, such as posture, indicate rejection, rather than inclusion, and the overall affective tone is neutral. Furthermore, when interacting as a couple, F2 and M2 entirely validate B2’s experience, paying attention to his emotional signals, while only partially validating them in the remaining three interaction phases, when their reactions to B2’s emotional signals are mostly unsuitable. There is some tension and covered hostility between F2 and M2, which lead to a fourth phase of observation, marked by a very low level of interaction and an insufficient duration. B2’s self-regulation is particularly effective when playing alone. The Global Family Alliance level was assigned the medium score of 15.

## Family 3: Medical History After B3’s Birth

B3 was born at 34 weeks with planned Cesarean section. Her weight at birth was 2,350 g. Two hours after birth, surgery was carried out to completely reposition B3’s intestines. The baby was then transferred to neonatal intensive care on mechanical ventilation. At 5 days B3 started sucking her mother’s milk, with a very high tolerance level. Meals were gradually increased, until, at 3 weeks, B3 reached complete feeding independence and parenteral nutrition was suspended. B3 remained in intensive care in the Neonatology and Neonatal Intensive Therapy Unit for 21 days, and was discharged with a weight of 2,670 g, with instructions to feed her M3’s milk every 3 h, from the breast or bottle. The family was not required to follow any intrusive medical procedure. Due to her general state and constant vomiting, the child was hospitalized again for 15 days at the age of 3 months.

## Family 3: LTP Procedure

During the various LTP phases B3 is rarely involved, she is disconnected from F3 and M3, almost as if she was somewhere else: she does not react to their inputs, and fixes on one single object, while her parents follow her in her activities. Both parents’ bodies clearly display unavailability to interact. They keep turning toward B3, watching her, talking about her and addressing her during their interaction phase. F3–B3 interaction is brief and lacks any kind of interaction and verbalisation. F3 holds B3’s shoe, but he does not even try to meet her gaze; he lets her play with the shoe, without ever interacting with her. When M3 leans forward to grasp a toy that is about to fall down, F3 uses this moment to sit back on his chair and let M3 take over. M3 tries to catch B3’s attention, while the latter keeps focusing on her shoes. M3 offers B3 multiple inputs, but the baby keeps avoiding her and self-excluding. Even when M3 takes B3’s face in her hands, the baby keeps staring down, looking for her shoes. M3 tries to change approach multiple times, yet no contact is established.

During the third configuration, F3 is visibly incapable of finding his space and/or interact with B3. Interaction takes place on a one-to-one basis, never involving all three parties at once. B3 focuses on her baby chair. Her parents do not seem to be able to adjust to her age and affective state. F3, in particular, under stimulates B3, whose attention and interest require continuous innovations, especially through multimodal input. Neither during the two-party interaction, nor during the three-party one is co-construction achieved, and there is no collaboration in carrying out any activity. Shared activities are brief and superficial. As regards the emotional atmosphere, M3 seems unable to understand B3’s status, as she constantly stimulates her, ignoring her unavailability signals. Shared affection moments never involve more than two parties. During the three-party playing phase parents perform different actions alternatively, without negotiating them. Distance is also managed in turns. Each parent follows his/her own activity pattern without interfering in the other’s, but no game is seamless. Throughout the entire playing phase B3 acts unavailable to her parents, with her body and gaze indicating detachment and avoidance. While interacting with F3, B3 is able to self-regulate. When interacting with M3, however, an almost methodical tendency to self-exclude is observed, as a reaction to M3 over-stimulation. The same is observed during the three-party interaction phase, when parents take it in turns to play with B3. B3’s alienation results in overstimulation by her parents, especially by M3, which only causes B3 to shut down further, self-exclude and focus on one object instead of her mother. Global Family Alliance level was assigned the medium-low score of 13.

## Discussion

Analysis of families’ interactions suggest maternal over-stimulation, which children respond to with prolonged withdrawal. Fathers, on the other hand, display significant difficulties in interacting with their babies with appropriate stimulations and understanding their emotional needs. These patterns of interaction may result in the child being increasingly passive and evasive. For these three families the interaction with the newborn was difficult from the beginning: the possibility of meeting their babies only through the incubator and the set of intrusive procedures (surgery, catheters, mechanical ventilation, and nasogastric tube) are experienced as painful for the baby (Carbajal et al., 2008) and result in a feeling of uncertainty and fragility surrounding the families (Montirosso et al., 2012).

Furthermore, during LTP observation, the marital space is sacrificed and the child is the organizing factor in the relationship between the parents. We believe that this may be linked to the fact that these children have absorbed all the energy of these fathers and mothers in their parental role, leaving no time or space for them to express their marital relationship. Further, over the course of the difficult months following the diagnosis and during the intensive care period, parents may experience the feeling of losing their child several times, leading them to over-stimulate them later on, because the baby’s reactions are considered a sign of liveliness. Moreover, it is likely that, being aware of the camera filming them, parents are eager to show the observers, and perhaps also themselves, how competent their child is, despite the difficulties he experienced.

The three couples engage in interactions, that, albeit over-stimulating and offset at times, are not excessive and/or pathological. Despite the numerous risk factors involved and partially thanks to the clinical support, these parents appear to have come to terms with their “real” child, as opposed to their idealized child, which means they have developed a number of tools to live with the babies, as they have faced the surgical operations, and the period of hospitalization in intensive care (Hugger, 2009). Indeed, only a few months after birth, these parents have the chance to interact with a lively newborn that they can see and touch. This aspect, together with the child’s positive reaction to surgery and medical treatment, give the parents hope, mitigating their fears of death and illness (Clarici et al., 2015; Tripani et al., 2015).

One of the most important limits of the study was the limited number of participants, as well as the possible correlated selection bias. Data are therefore presented as a case study, as the number of participants does not allow the analysts to make any inference about the entire population; at the same time, however, our results provide an insight into the possible uses of the employed instrument to collect behavioral data, while isolating a series of tendencies in families’ interactions when baby’s surgery is involved.

## Concluding Remarks

We believe that the investigation carried out on these three clinical cases may serve as a basis for the development of a tailored psychological treatment for parents-to-be receiving a prenatal diagnosis (e.g., video-feedback-base psychotherapeutic treatments see Fukkink, 2008), and even as a starting point for further longitudinal research on at-risk pediatric populations.

## Author Contributions

SP designed the study, drafted a first version of work and provided a final approval of the manuscript. AT designed of the study, acquired the data and approved a final version of the manuscript. MM analyzed the data, proposed a critical revision of the work and approved final approval of the manuscript. RG acquired and critically interpreted the data and provide a final approval of the last version of the manuscript. AC supervised a critical revision of the work and proposed the final approval of the version to be published.

## Conflict of Interest Statement

The authors declare that the research was conducted in the absence of any commercial or financial relationships that could be construed as a potential conflict of interest.
